# Synthesis and Stability of Hydrogen Storage Material Aluminum Hydride

**DOI:** 10.3390/ma14112898

**Published:** 2021-05-28

**Authors:** Wenda Su, Fangfang Zhao, Lei Ma, Ruixian Tang, Yanru Dong, Guolong Kong, Yu Zhang, Sulin Niu, Gen Tang, Yue Wang, Aimin Pang, Wei Li, Liangming Wei

**Affiliations:** 1 Key Laboratory for Thin Film and Microfabrication Technology of the Ministry of Education, Department of Microelectronics and Nanoscience, School of Electronics Information and Electrical Engineering, Shanghai Jiao Tong University, Dong Chuan Road No. 800, Shanghai 200240, China; wendasu@sjtu.edu.cn (W.S.); zhaofangfang@sjtu.edu.cn (F.Z.); leima00@sjtu.edu.cn (L.M.); sugarapple@sjtu.edu.cn (R.T.); yanerdyr@sjtu.edu.cn (Y.D.); KongGuoLong@sjtu.edu.cn (G.K.); zhyurainy@sjtu.edu.cn (Y.Z.); niu_sulin@sjtu.edu.cn (S.N.); 2 Science and Technology on Aerospace Chemical Power Laboratory, Hubei Institute of Aerospace Chemotechnology, Xiangyang 441003, China; tanggen518@126.com (G.T.); wang__yue21@126.com (Y.W.)

**Keywords:** AlH_3_, hydrogen storage, high energy material, stability

## Abstract

Aluminum hydride (AlH_3_) is a binary metal hydride with a mass hydrogen density of more than 10% and bulk hydrogen density of 148 $\mathrm{kg}\;H_{2}/m^{3}$. Pure aluminum hydride can easily release hydrogen when heated. Due to the high hydrogen density and low decomposition temperature, aluminum hydride has become one of the most promising hydrogen storage media for wide applications, including fuel cell, reducing agents, and rocket fuel additive. Compared with aluminum powder, AlH_3_ has a higher energy density, which can significantly reduce the ignition temperature and produce H_2_ fuel in the combustion process, thus reducing the relative mass of combustion products. In this paper, the research progress about the structure, synthesis, and stability of aluminum hydride in recent decades is reviewed. We also put forward the challenges for application of AlH_3_ and outlook the possible opportunity for AlH_3_ in the future.

## 1. Introduction

Aluminum hydride (AlH_3_) has great potential applications in rocket fuel and fuel cell due to its high combustion heat and high hydrogen content [1,2,3]. The bulk hydrogen density of AlH_3_ is 148 $\mathrm{kg}\;H_{2}/m^{3}$ (more than twice of liquid hydrogen), and the weight hydrogen density is more than 10%, which meets the requirements of the U.S. Department of Energy (DOE) for hydrogen and energy storage materials [4,5,6,7,8,9]. AlH_3_ also has the advantages of low reaction heat and rapid hydrogen release rate. However, AlH_3_ is unstable and easy decomposition occurs at room temperature, and it can react with water vapor and oxygen in the environment, resulting in the release of hydrogen. Therefore, how to improve the stability of AlH_3_ has become the key problem to be solved in the practical application of AlH_3_ [10].

In recent years, researchers are committed to breaking through the bottleneck problems in the practical application of AlH_3_, including improving the synthesis method [11,12,13,14], coating or doping of AlH_3_ [15,16,17], and providing a deep understanding of the thermodynamics and kinetics of AlH_3_ [18,19,20]. This review focuses on the recent studies on AlH_3_. In this review, we first introduce the physical and chemical properties of aluminum hydride, including crystal structure and thermal decomposition reaction kinetics. Next, the common synthesis methods of aluminum hydride are described. Last, we put forward the methods to improve the stability of aluminum hydride and outlook the possible opportunities for application of AlH_3_ in the future.

## 2. Physical and Chemical Properties

**Figure 1:**
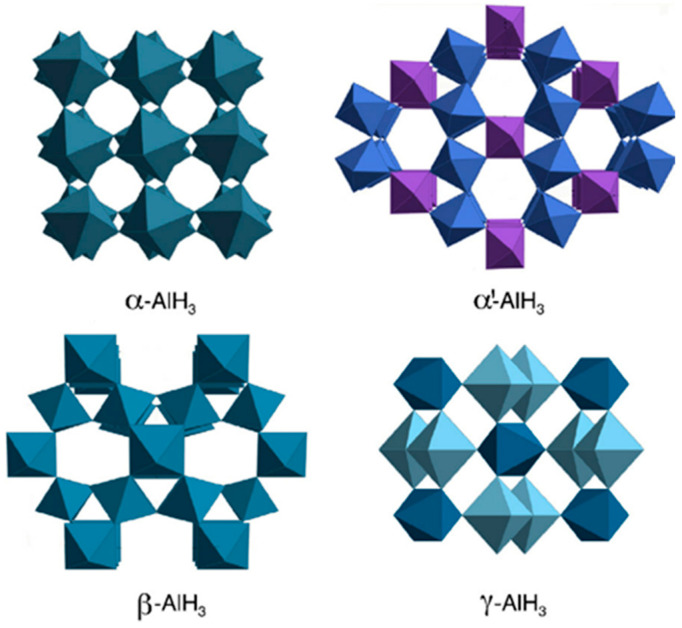
Structures of AlH_3_ polymorphs of $\alpha -{\mathrm{AlH}}_{3}$, $\alpha^{\prime }-{\mathrm{AlH}}_{3}$, $\beta -{\mathrm{AlH}}_{3}$, $\gamma -{\mathrm{AlH}}_{3}$. Reprinted with permission from Ref. [10]. Copyright 2009 Elsevier.

There are at least seven different crystal structures of AlH_3_ due to the different synthesis routes and reaction conditions (mainly referring to the reaction time and temperature) [21,22]. Brower et al. [23] first synthesized α phase of non-solvated aluminum hydride, and then successfully synthesized six other AlH_3_ polymorphs: $\alpha^{\prime },\;\beta ,\;\gamma ,\;\delta ,\;\varepsilon \;\mathrm{and}\;\zeta$. It has been proved by experiment and theory that the α crystal phase is the most stable one, followed by the β and γ crystal phase [24]. Figure 1 shows several common structures of AlH_3_ polymorphs [10]. The α phase belongs to the trigonal space group ($R\bar{3}c$), with the lattice parameters of *a* = 4.449 Å and *c* = 11.804 Å [25]. The structure is composed of alternating planes of H and Al atoms, which are stacked perpendicularly to the *C*-axis. There are six H atoms around each Al atom to form regular octahedral coordination. Because the Al atom has only three bonding electrons, two Al atoms form the Al-H-Al electron deficient bridge bond by sharing one electron of H atom. The structure is composed of an Al-H-Al bridge bond, which connects the atoms in the whole unit cell into a relatively stable whole. Therefore, the α phase has good chemical stability in macroscopic view. The crystal parameters of the α phase are listed in Table 1. The β phase belongs to the cubic space group (Fd$\bar{3}$m), with the lattice parameters of *a* = 9.004 Å [26]. The γ phase belongs to the orthorhombic space group (Fd$\bar{3}$m), with the lattice parameters of *a* = 5.3806(1) Å, *b* = 7.3555(2) Å, *c* = 5.77509(5) Å [21]. $\beta -{\mathrm{AlH}}_{3}$ and $\gamma -{\mathrm{AlH}}_{3}$ will spontaneously convert to $\alpha -{\mathrm{AlH}}_{3}$ at around 100 °C [27], and the reaction heats of polymorph transformations are: 1.5 kJ/mol for the $\beta -{\mathrm{AlH}}_{3}$ → $\alpha -{\mathrm{AlH}}_{3}$ transition and 2.8 kJ/mol for the $\gamma -{\mathrm{AlH}}_{3}$ → $\alpha -{\mathrm{AlH}}_{3}$ transition [24]. According to the reaction heat of the polymorph transformation, $\alpha -{\mathrm{AlH}}_{3}$ is proved to be the most stable phase. Therefore, only the α phase has a practical application value. In this review, all aluminum hydride involved is α phase except for special indication.

**Figure 2:**
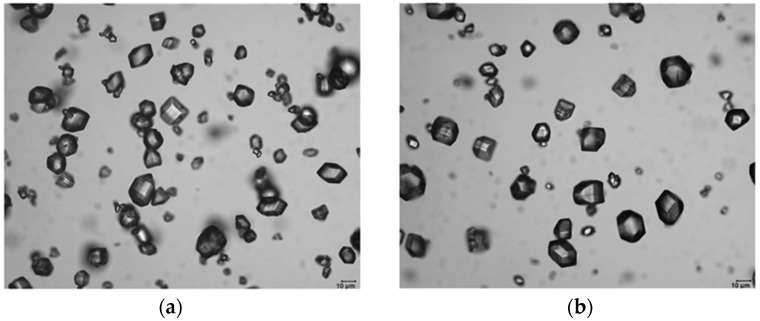
Optical microscopy pictures of $\alpha -{\mathrm{AlH}}_{3}$. (**a**,**b**) are optical microscope pictures of $\alpha -{\mathrm{AlH}}_{3}$ at different magnification. Reprinted with permission from Ref. [28]. Copyright 2009 WILEY-VCH Verlag GmbH & Co. KGaA, Weinheim.

**Figure 3:**
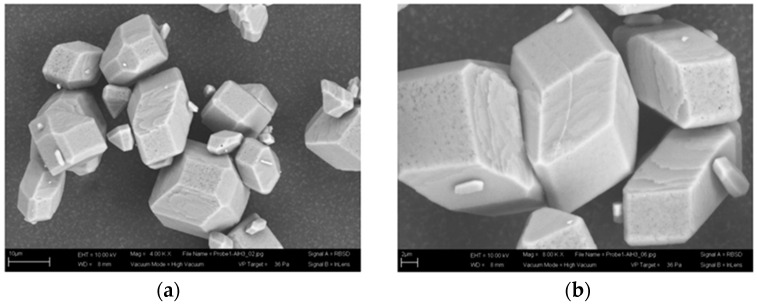
SEM pictures of $\alpha -{\mathrm{AlH}}_{3}$. (**a**,**b**) are SEM pictures pictures of $\alpha -{\mathrm{AlH}}_{3}$ at different magnification. Reprinted with permission from Ref. [28]. Copyright 2009 WILEY-VCH Verlag GmbH & Co. KGaA, Weinheim.

$\alpha -{\mathrm{AlH}}_{3}$ is a white nonvolatile hexagonal crystal with a density of 1.477 $g/{\mathrm{cm}}^{3}$, the theoretical hydrogen content of 10.08%, insoluble or slightly soluble in ether, soluble in tetrahydrofuran, and inert to hydrazine [29,30]. When exposed to water, the pure $\alpha -{\mathrm{AlH}}_{3}$ hydrolyzes slowly, and takes several hours for complete hydrolyses [31,32].$\;\alpha -{\mathrm{AlH}}_{3}$ can be stored for several months at room temperature, and it begins to decompose at 100 °C, releasing hydrogen and forming aluminum [33]. The optical and scanning electron microscopy pictures of $\alpha -{\mathrm{AlH}}_{3}$ are reported (as shown in Figure 2 and Figure 3). It can be seen that $\alpha -{\mathrm{AlH}}_{3}$ is a dense and transparent crystal with a good crystal plane and crystal edge configuration [28].

## 3. Synthesis Methods

### 3.1. Liquid Phase Synthesis Methods

In 1942, Stecher and Wiberg [34] first prepared ${\mathrm{AlH}}_{3}\cdot 2N{\left({\mathrm{CH}}_{3}\right)}_{3}$ with low yield and purity by liquid phase synthesis. A few years later, Finholt et al. [35] prepared a solution of AlH_3_ in ether by the reaction of LiAlH_4_ and AlCl_3_. The chemical equation of the reaction is:

(1) $$3{\mathrm{LiAlH}}_{4}+{\mathrm{AlCl}}_{3}\;\overset{\mathrm{ether}}{\to }\;4{\mathrm{AlH}}_{3}+3\mathrm{LiCl}$$

Finholt et al. [35] also proposed another reaction route for the preparation of AlH_3_: the reaction of LiH and AlCl_3_ in ether solution. The chemical equation of the reaction is as follows:

(2) $$3\mathrm{LiH}+{\mathrm{AlCl}}_{3}\;\overset{\mathrm{ether}}{\to }{\;\mathrm{AlH}}_{3}+3\mathrm{LiCl}$$

For these two different reaction pathways, Finholt et al. [35] found that although Reaction (2) was simpler, Reaction (1) was more stable and rapid. At the same time, the target product of Reaction (2) can easily continue to react to form LiAlH_4_, so Reaction (1) was considered to be a better choice. The final product prepared by Finholt et al. was ${\mathrm{AlH}}_{3}\cdot 0.3\left[{\left(C_{2}H_{5}\right)}_{2}O\right]$ with a yield of 85%. However, due to the presence of a certain amount of ether in the final product, how to remove the ether without loss of hydrogen becomes the focus of the next step.

In 1955, Chizinsky [36] prepared AlH_3_ without ether solvent for the first time. The AlH_3_ ether solution prepared by the usual method was filtered into inert liquid (pentane and ligroin), and then dried under a high vacuum for 12 h. However, Chizinsky’s experimental results have not been effectively repeated. In 1975, Brower et al. [23] published a summary on the preparation of AlH_3_. They synthesized seven different polymorphs of AlH_3_. The synthesis method is similar to that of Stecherand Wiberg [34] and Finholt et al. [35], namely the metathesis reaction between LiAlH_4_ and AlCl_3_ (Reaction (3)), and then the ether is removed by heating the mixed solution of ether and toluene with the good solubility of ether in toluene (Reaction (4)).

(3) $$4{\mathrm{LiAlH}}_{4}+{\mathrm{AlCl}}_{3}+{\mathrm{Et}}_{2}O\;\to \;4{\mathrm{AlH}}_{3}\cdot {\mathrm{nEt}}_{2}O+3\mathrm{LiCl}↓+{\mathrm{LiAlH}}_{4}+{\mathrm{Et}}_{2}O$$

(4) $$4{\mathrm{AlH}}_{3}\cdot {\mathrm{Et}}_{2}O+{\mathrm{LiAlH}}_{4}+{\mathrm{LiBH}}_{4}+{\mathrm{Et}}_{2}O\underset{5h}{\overset{65℃}{=}}\;4{\mathrm{AlH}}_{3}+{\mathrm{Et}}_{2}O↑+{\;\mathrm{LiAlH}}_{4}↓+\;{\mathrm{LiBH}}_{4}↓$$

Brower et al. [23] found that the pure AlH_3_ etherate would decompose and result in loss of hydrogen when heated in vacuum, but if there was excessive LiAlH_4_, the ether in AlH_3_ etherate could be removed. They also found that excess LiAlH_4_ can decrease the time and temperature of desolvation. The mixed use of LiAlH_4_ and LiBH_4_ can further reduce the temperature and time of desolvation reaction. However, when only LiBH_4_ exists and LiAlH_4_ is absent, desolvation cannot be achieved [23]. When the molar ratio of LiAlH_4_: AlH_3_: LiBH_4_ is 1:4:1, $\gamma -{\mathrm{AlH}}_{3}$ is prepared [23]. Brower et al. [23] carried out X-ray analysis of the prepared γ—AlH_3_ and found that it has the same X-ray powder pattern as the sample reported by Chizinsky. Therefore, Chizinsky’s experimental results are considered to be caused by accidental use of excessive LiAlH_4_.

Different polymorphs of AlH_3_ can be prepared by slightly adjusting Reactions (3) and (4). $\gamma -{\mathrm{AlH}}_{3}$ was prepared by reducing the molar ratio of LiBH_4_ and reacting at low temperature, while $\varepsilon -{\mathrm{AlH}}_{3}$ and $\delta -{\mathrm{AlH}}_{3}$ were prepared in the presence of a small amount of water. These nonsolvated phases are not considered to be converted into $\alpha -{\mathrm{AlH}}_{3}$, and their thermal stability is much worse than that of $\alpha -{\mathrm{AlH}}_{3}$ [23].

Brower et al. [23] also designed a process to prepare a large number of microcrystalline $\alpha -{\mathrm{AlH}}_{3}$: ether solution was prepared according to Reaction (3), and then desolvent in toluene (or benzene) using a crystallization flask with a fractionator. After that, the researchers proposed a variety of liquid-phase synthesis methods of AlH_3_. The researchers found that etherated or pure AlH_3_ could be prepared by the reaction of binary hydrides or tetrahydroaluminates (aluminates) of the first and second groups with Bronsted-Lewis acids such as AlCl_3_ [37].

In recent years, researchers have improved the wet synthesis process. Bulychev et al. [38] synthesized unsolvated AlH_3_ by the reaction of AlBr_3_ or sulfuric acid with LiAlH_4_ in pure toluene or in toluene containing 5–10 wt% diethyl ether. However, the unsolvated AlH_3_ is not a high-purity crystal, and the concentrated sulfuric acid used in the reaction process is comparatively dangerous.

Lacina et al. [39] carried out vacuum distillation for the mixed solution of ether compounds, and successfully obtained micrometer grade α—AlH_3_ by using a variable temperature solvent crystallization method. Graetz et al. [40] reported a low-temperature, low-pressure, reversible reaction using titanium-doped aluminum powder and triethylenediamine (TEDA) to regenerate AlH_3_. They first prepared AlH_3_ by a traditional method, then doped it with β-TiCl_3_. After that, the samples were dried and decomposed in vacuum to produce active aluminum powder containing titanium. AlH_3_-TEDA can be regenerated by the reaction of the active aluminum powder with hydrogen and TEDA in THF solution at a lower temperature and pressure.

Although the liquid-phase synthesis method of AlH_3_ has been mature and perfect, the liquid-phase synthesis method itself has some disadvantages, such as being unsafe, consuming a lot of solvents, involving a complex follow-up processing, and so on. Therefore, the new synthesis method without solvent has attracted the attention of researchers.

### 3.2. Dry Synthesis

As mentioned above, the synthesis of AlH_3_ in solvents requires a lot of solvents, and most of these solvents are expensive and toxic, so the method of synthesizing AlH_3_ without liquid phase solvents has been developed. One way of dry synthesis is to make aluminum react with hydrogen directly under high pressure to produce AlH_3_. Baranowaski and Tkacz [41] have systematically measured the equilibrium between gaseous hydrogen and solid aluminum hydride in the temperature range of 100–150 °C and pressure range of 0–12 kPa. The results show that AlH_3_ can be synthesized only when the temperature exceeds 140 °C [41]. Appel and Frankel [42] bombarded pure aluminum target samples with deuterons. The X-ray diffraction patterns of the samples showed that a new phase was formed. By comparison, it was confirmed that AlH_3_ was obtained on the surface of the aluminum target [42].

**Figure 4:**
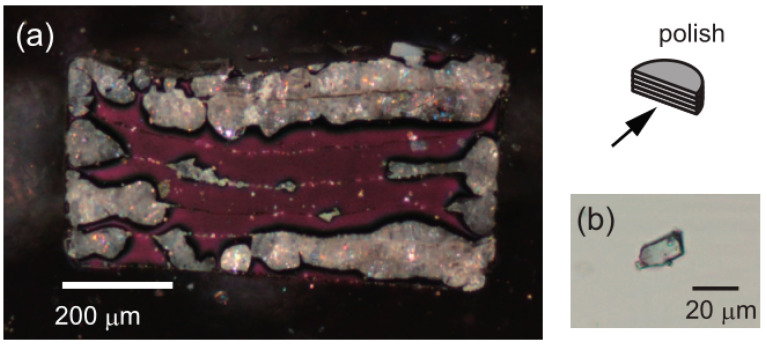
(**a**) Polarized micrograph of a longitudinal section of a recovered disk sample hydrogenated at 650 °C and 10.0 GPa for 24 h. (**b**) Transmitted light micrograph of the obtained AlH3 single crystal. Reprinted with permission from Ref. [43]. Copyright 2008 AIP Publishing.

Saitoh et al. [43] prepared AlH_3_ via reacting aluminum with hydrogen at 650 °C and 10.0 GPa for 24 h. They measured the morphology change of aluminum, as shown in Figure 4, and found that the surface of aluminum is covered with a layer of white particles, which are colorless and transparent crystals under the transmission light microscope. The white particles were confirmed to be AlH_3_ by powder X-ray diffraction.

**Figure 5:**
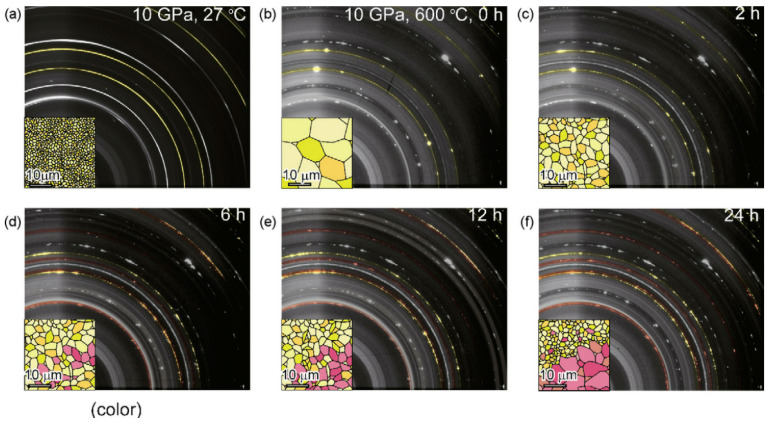
X-ray diffraction images of the sample during the hydrogenation. (**a**) Complete Debye rings from aluminum at 10 GPa, 27 °C. (**b**) Bragg spots from aggregated aluminum (colored in yellow). This image was taken immediately after the sample was heated to 600 °C at 10 GPa. (**c**–**f**) Diffraction images taken after 2 h, 6 h, 12 h, and 24 h heat treatment at 10 GPa, 600 °C. Reprinted with permission from Ref. [44]. Copyright 2010 Elsevier.

Saitoh et al. [44] studied the reaction process of aluminum and hydrogen at 10 GPa and 600 °C by in situ X-ray diffraction. As shown in Figure 5, the X-ray diffraction images of the samples are collected every 10 min during the hydrogenation process [44]. The yellow and red particles represent Al and AlH_3_ particles, respectively. Saitoh et al. found that the growth process of AlH_3_ single crystal will go through three stages: self-pulverization of aluminum (with the increase of reaction time, it is found that the size of aluminum Bragg lattice decreases), hydrogenation of pulverized aluminum (X-ray diffraction image shows a new Bragg lattice), and solid-state grain growth of AlH_3_ [44].

The direct reaction of aluminum and hydrogen under high pressure to produce AlH_3_ often requires strict reaction conditions and low yield, so this kind of method is not considered as a good choice [45]. It is considered that mechanical ball milling is a simple and feasible method for the synthesis of metal compound hydrides. Mechanical ball milling has been used to synthesize Mg(AlH_4_)_2_ [46,47], Ca(AlH_4_)_2_ [48,49], LiMg(AlH_4_)_2_ [50], and so on. Brinks et al. [22] first proposed the mechanical ball milling method to prepare AlD_3_. They prepared AlD_3_ by using the mixed ball milling of 3LiAlD_4_ and AlCl_3_. Brinks et al. [22] found that in addition to AlD_3_ (α and $\alpha^{\prime }$), the mixture of LiCl and Al was also produced in planetary milling at room temperature, while only AlD_3_ and LiCl were produced in freezing ball milling at 77 K. Sartori et al. [51] found that AlH_3_ can be prepared by cryomilling aluminium halides and alanate. They also found that the yield of AlH_3_ can be increased by using 3LiAlD_4_ + AlBr_3_ and 3NaLiAlD_4_ + AlCl_3_ compared with using 3LiAlD_4_ + AlCl_3_.

**Figure 6:**
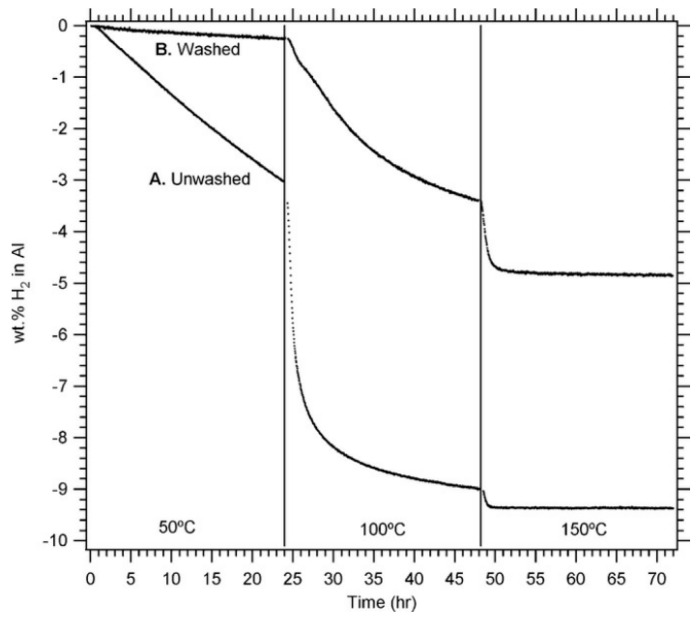
Hydrogen desorption data from (**A**) unwashed and (**B**) washed samples cryogenically milled for 1 h with a 2:1 LiCl:AlH_3_ volume ratio. Reprinted with permission from Ref. [52]. Copyright 2009 Elsevier.

Paskevicius et al. [52] studied the structure of AlH_3_ prepared by mechanical grinding, and found that some $\beta -{\mathrm{AlH}}_{3}$ and $\gamma -{\mathrm{AlH}}_{3}$ were formed in the product. After ball milling, LiCl in the product was removed via washing with nitromethane/AlCl_3_ solution, and AlH_3_ separated from the by-product was obtained. However, the AlH_3_ nanoparticles adversely reacted with the nitromethane/AlCl_3_ solution. The hydrogen desolvation kinetics of the washed samples were significantly hindered compared to the unwashed samples [52]. The hydrogen desorption ability of the washed sample is only half of that unwashed sample, as shown in Figure 6.

**Figure 7:**
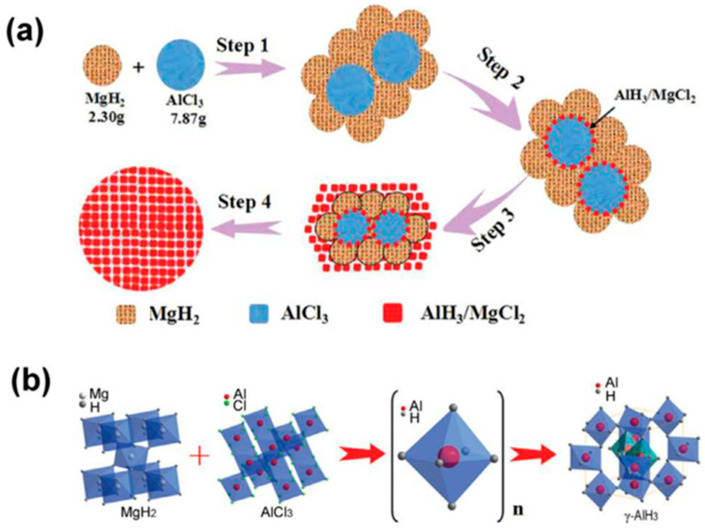
(**a**) The pathway of the mechanically activated reaction between MgH_2_ and AlCl_3_. (**b**) Mechanism of the solid state synthesis of nano-sized $\gamma -{\mathrm{AlH}}_{3}$. Reprinted with permission from Ref. [56]. Copyright 2021 Elsevier.

Duan et al. [53] proposed a cost-effective solid-state reaction grinding method with cheap metal hydride and aluminum chloride as the starting reagents to synthesize AlH_3_. They found that nano-sized $\gamma -{\mathrm{AlH}}_{3}$ can be synthesized by reactive milling the AlCl_3_ and MgH_2_ nanocrystalline. According to XRD, NMR, and TEM analysis, the average size of the as-synthesized $\gamma -{\mathrm{AlH}}_{3}$ phase was estimated to be around 8.5 nm [53]. Duan et al. [54] also found that MgH_2_ can be stably converted to $\gamma -{\mathrm{AlH}}_{3}$ in the process of grinding the MgH_2_/AlCl_3_ mixture. Further studies by TEM, SEM, and NMR showed that the amorphous intermediate (AlH_6_)_n_ was synthesized preferentially and recrystallized to $\gamma -{\mathrm{AlH}}_{3}$ at the end of the reaction [54]. Surface nanocrystallization is considered to be a key step in mechanochemical synthesis of AlH_3_, and the synthesis process can be shown in Figure 7 [55].

Hlova et al. [57] studied the mechanical grinding process for the synthesis of AlH_3_ from LiH and AlCl_3_ at room temperature. The intermediates and final products were characterized by powder X-ray diffraction and solid-state ^27^Al NMR spectroscopy. The experimental results show that adding AlCl_3_ slowly during ball milling can prevent the formation of unnecessary intermediate products. At the same time, if the reaction is carried out at 3 MPa hydrogen pressure, the final products are not easy to decompose.

Although the mechanical ball milling method has its unique advantages to synthesize AlH_3_, it is difficult to be used in large-scale production due to its complex preparation process, high reaction conditions (usually at a low temperature or liquid nitrogen), mixed crystal phase of reaction products, and difficult purification.

### 3.3. Other Synthetic Methods

In the process of synthesizing AlH_3_ by mechanical ball milling, the reactants are heated unevenly, and other complex crystal phases are often produced. Therefore, other synthetic methods of AlH_3_ have been proposed in recent years. Supercritical technology is a new experimental method and plays an increasingly important role in chemical separation and synthesis. McGrady [58] prepared AlH_3_ by supercritical technology. In a pressure vessel without water and oxygen, aluminum powder and 2% catalyst (TiCl_3_) were mixed evenly, and liquid carbon dioxide (89 MPa) and hydrogen (50 MPa) were introduced, heated to 60 °C to reach supercritical state, stirred at a rate of 150 r/min for 1 h, and then cooled to room temperature to obtain gray powder AlH_3_. However, due to the limitation of experimental conditions, supercritical technology cannot be applied to the actual production of AlH_3_.

**Figure 8:**
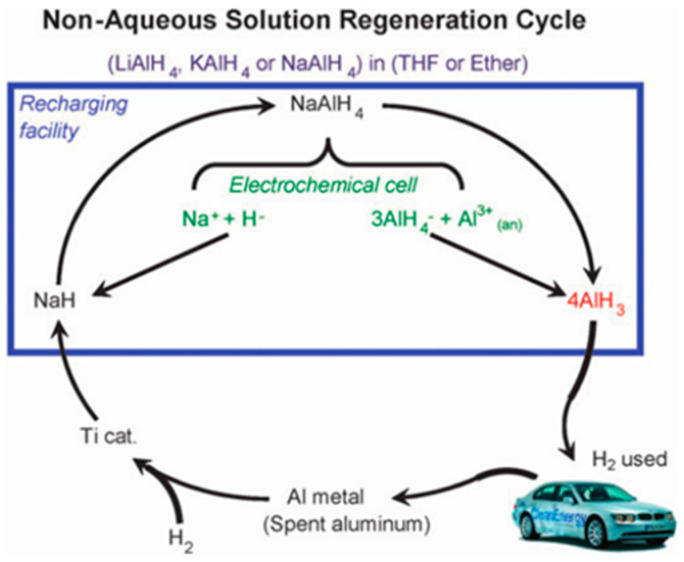
Proposed reversible fuel cycle for alane. Reprinted with permission from Ref. [56]. Copyright 2021 Elsevier.

Zidanet al. [59] synthesized $\alpha -{\mathrm{AlH}}_{3}$ by an electrochemical method for the first time: AlH_3_·nTHF adduct was obtained by electrolysis with THF as solvent, NaAlH_4_ as electrolyte, aluminum as anode, and platinum as cathode. Due to the strong binding force of AlH_3_·nTHF, AlH_3_·TEA was synthesized by introducing triethylamine (TEA). Then, pure α—AlH_3_ was obtained by heating the AlH_3_·TEA solution in vacuum [59]. This method can realize the reversible cycle of material regeneration, as shown in Figure 8.

## 4. Stability

There are at least seven different crystal structures in AlH_3_, among which $\alpha -{\mathrm{AlH}}_{3}$ is the most stable. $\alpha -{\mathrm{AlH}}_{3}$ will decompose to form aluminum and hydrogen, as in Reaction (5) [60].

(5) $$\alpha -{\mathrm{AlH}}_{3}\to \mathrm{Al}+\frac{3}{2}H_{2}↑$$

(6) $$\alpha^{\prime }-{\mathrm{AlH}}_{3}/\beta -{\mathrm{AlH}}_{3}/\gamma -{\mathrm{AlH}}_{3}\to \alpha -{\mathrm{AlH}}_{3}$$

(7) $$\alpha^{\prime }-{\mathrm{AlH}}_{3}/\gamma -{\mathrm{AlH}}_{3}\to \mathrm{Al}+\frac{3}{2}H_{2}↑$$

**Figure 9:**
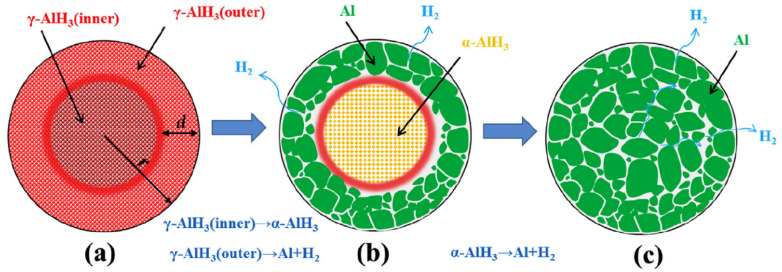
Schematic picture showing the outer layers and the inner parts of $\gamma -{\mathrm{AlH}}_{3}$ particle behaving with different decomposition mechanisms: (**a**) as-synthesized $\gamma -{\mathrm{AlH}}_{3}$, (**b**) after partial decomposition, (**c**) after full decomposition. Reprinted with permission from Ref. [62]. Copyright 2017 Elsevier.

Sinke et al. [9] investigated the thermodynamic properties of $\alpha -{\mathrm{AlH}}_{3}$, and they found that the enthalpy of formation of $\alpha -{\mathrm{AlH}}_{3}$ at room temperature was −11.4 kJ/mol. $\alpha^{\prime }-{\mathrm{AlH}}_{3}$, $\beta -{\mathrm{AlH}}_{3}$, and $\gamma -{\mathrm{AlH}}_{3}$ are unstable and will transform into $\alpha -{\mathrm{AlH}}_{3}$ during heating (Reaction (6)) [27]. During the heating process, the direct decomposition of $\gamma -{\mathrm{AlH}}_{3}$ and $\alpha^{\prime }-{\mathrm{AlH}}_{3}$ into Al and H_2_ was also observed (Reaction (7)) [61]. Gao et al. [62] found that the outer part of $\gamma -{\mathrm{AlH}}_{3}$ particles inclines to decompose directly, but the inner part will first convert to $\alpha -{\mathrm{AlH}}_{3}$, and then decompose to Al and H_2_, as shown in Figure 9.

**Figure 10:**
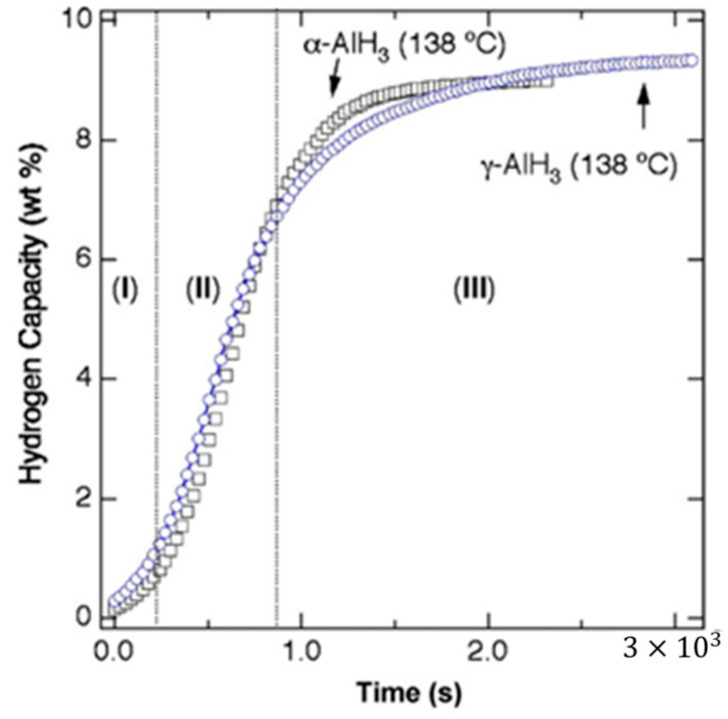
High temperature isothermal decomposition curves from α and γ-AlH_3_ starting materials at 138 °C showing (**I**) induction period (**II**), acceleratory period, and (**III**) decay period. Reprinted with permission from Ref. [63]. Copyright 2007 Elsevier.

Graetz et al. [63] studied the isothermal decomposition process of AlH_3_, and found that the isothermal decomposition curve of AlH_3_ was sigmoid, including induction period, acceleration period, and decay period, as shown in Figure 10. Graetz et al. [63] also measured that the activation energy of the $\alpha -{\mathrm{AlH}}_{3}$ decomposition reaction with a small grain size of 102 kJ/mol, which is similar to that of the $\alpha -{\mathrm{AlH}}_{3}$ decomposition reaction with a large grain size measured by Gabis et al. [64]. This indicates that the grain size has no significant effect on the activation energy of the AlH_3_ decomposition reaction. However, the above measured values are significantly lower than those of the stabilized $\alpha -{\mathrm{AlH}}_{3}$ (157 kJ/mol) reported by Herley et al. [65]. This indicates that the formation of $\alpha -{\mathrm{AlH}}_{3}$ surface oxide can improve its stability. The enthalpy of dehydrogenation of $\alpha -{\mathrm{AlH}}_{3}$ determined by DSC (Differential Scanning Calorimetry) is 5.7–6.6 kJ/mol of H_2_ [66]. Therefore, AlH_3_ is easy to release hydrogen at elevated temperatures.

As mentioned above, AlH_3_ has poor stability and is easy to decompose to produce hydrogen when the temperature rises, which makes the storage and transportation of AlH_3_ to have high risks. The structure, thermodynamics, kinetics, and synthesis methods of AlH_3_ have been comprehensively and summarized in detail in the latest several reviews about AlH_3_ [33,56,67]. However, the content of improving the stability of AlH_3_ is not much involved. In this chapter, we summarize and discuss the latest research papers on improving the stability of AlH_3_. The key to inhibit the decomposition of AlH_3_ is how to inhibit the appearance of the induction period [63]. At present, the main methods to stabilize AlH_3_ are surface passivation, doping stabilization, and surface coating.

### 4.1. Surface Passivation Method

**Figure 11:**
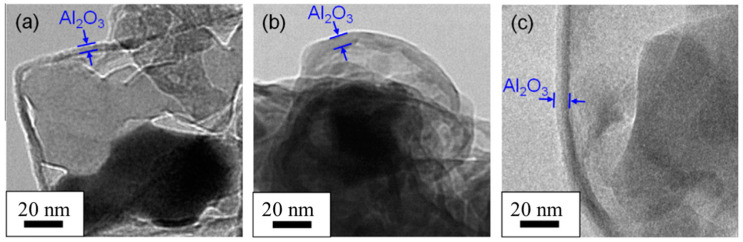
The TEM images of Al_2_O_3_ films on AlH_3_ particles; (**a**) without exposure to air, (**b**) 1 day after exposure to air, and (**c**) 7 days after exposure to air. Reprinted with permission from Ref. [5]. Copyright 2013 Elsevier.

Nakagawa et al. [5] observed the dehydrogenation and decomposition process of $\alpha -{\mathrm{AlH}}_{3}$ by in situ transmission electron microscopy. It was found that with the increase of storage time, the thickness of the surface Al_2_O_3_ layer increased, and Al grew at the interface of two phases, as shown in Figure 11. The results depict that the existence of alumina film can significantly delay the dehydrogenation of $\alpha -{\mathrm{AlH}}_{3}$. At the same time, the dehydrogenation temperature is independent of the thickness of the alumina film, which indicates that the introduction of the alumina film on the surface of AlH_3_ is a feasible method to inhibit the decomposition. In the surface passivation method, dilute acid, buffer solution, organic matter immersion, and heat treatment are used to remove the unstable impurity phase, treat the surface of $\alpha -{\mathrm{AlH}}_{3}$, quench the active sites, and form a dense Al_2_(OH)_3_ or Al_2_O_3_ layer to achieve the purpose of stabilization [5].

**Figure 12:**
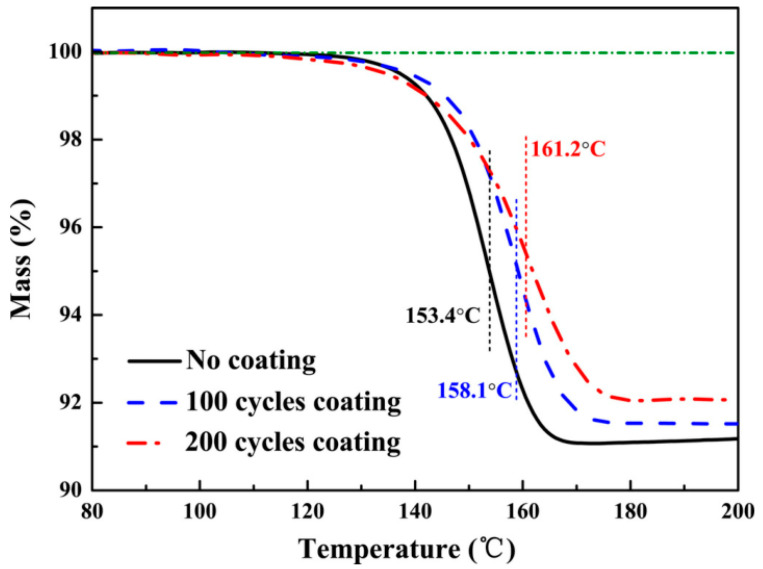
(Color online) TG curves of AlH_3_ and AlH_3_@Al_2_O_3_ samples at the heating rate of 2 °C/min. Reprinted with permission from Ref. [68]. Copyright 2017 American Vacuum Society.

Chen et al. [68] passivated a layer of nano-Al_2_O_3_ film on the surface of $\alpha -{\mathrm{AlH}}_{3}$ by atomic layer deposition. The hydrogen retention capacity of passivated particles was four times higher than that of untreated samples. At the same time, the hydrogen release speed of the passivated AlH_3_ samples was almost the same as that of the untreated samples, as shown in Figure 12. The dehydrogenation rate of passivated AlH_3_ particles was almost the same as that of untreated samples, which indicates that this is a practicable technology to stabilize AlH_3_ without sacrificing its energy release ability [68].

### 4.2. Doping Stabilization Method

The doping stabilization method is different from the surface passivation method. The doping method is to introduce metal ions or stabilizers in the synthesis stage of AlH_3_, and modify the crystal structure and defects of AlH_3_ to stabilize AlH_3_. Bulychev et al. [69] used LiAlH_4_·Mg(AlH_4_)_2_ and LiCI·Mg(AlH_4_)_2_ as modifying additives to increase the stability of AlH_3_. According to the data of element phase and X-ray phase analysis, it was found that the lattice parameters of $\alpha -{\mathrm{AlH}}_{3}$ increased after modification; Mg atoms entered into the lattice of $\alpha -{\mathrm{AlH}}_{3}$; and the morphology of the sample changed from cubic to spherical with a smooth edge [69]. The results of the hydrogen evolution rate test at 110 °C show that the thermal stability of modified $\alpha -{\mathrm{AlH}}_{3}$ is significantly improved.

**Figure 13:**
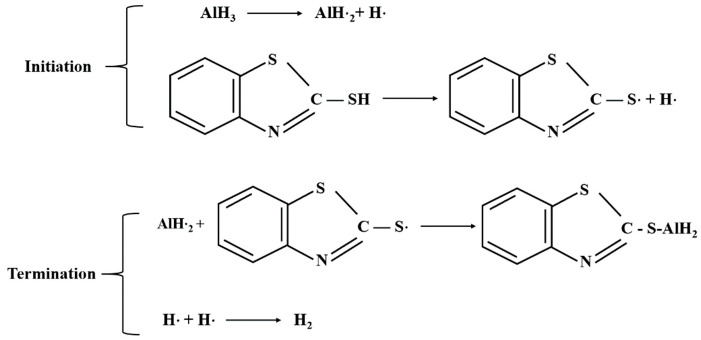
Stabilization mechanism of 2-Mercaptobenzothiazole (MBT) and $\alpha -{\mathrm{AlH}}_{3}$.

Ardis and Natoli [70] consider that heat, light, radiation, and other factors would cause the reaction of $\alpha -{\mathrm{AlH}}_{3}$ radicals, which would lead to the self decomposition of $\alpha -{\mathrm{AlH}}_{3}$. Therefore, the researchers tried to improve the stability by adding free radical inhibitors. Ardis et al. [70] added free radical inhibitors such as MBT and PTA into the synthesis process of $\alpha -{\mathrm{AlH}}_{3}$ (as shown in Figure 13) to obtain the modified samples. The stability test showed that the decomposition rate of the modified samples decreased from 7.5% to 0.6% after 17 days of storage. However, free radical scavengers need to be decomposed to generate active radicals, and then combine with hydrogen radicals, which will lead to the hydrogen radicals from AlH_3_ that have already combined with themselves to generate hydrogen before combining with scavengers. Therefore, the possibility of a non-radical reaction between 2-mercaptobenzothiazole and AlH_3_ needs further investigation. The doping stabilization method can significantly improve the stability of AlH_3_, but other elements introduced by doping may reduce the energy and purity of AlH_3_, affecting its compatibility and safety.

### 4.3. Surface Coating Method

Although the above two methods can significantly improve the stability of AlH_3_, they will affect the hydrogen release efficiency, compatibility, and thermal performance of AlH_3_. Researchers tend to use the surface coating method, which has little effect on the physical and chemical properties of AlH_3_, to stabilize AlH_3_.

Matzek et al. [71] reported a method of coating AlH_3_ with diphenylacetylene. AlH_3_ was washed with a benzene solution containing diphenylacetylene and then dried in nitrogen. It is found that the thermal stability of AlH_3_ increases with the increase of coating amount. It takes 13 days for uncoated AlH_3_ to decompose 1% at 60 °C, while it takes 29 days for AlH_3_ containing 2.5% diphenylacetylene. At the same time, magnesium-doped AlH_3_ coated with diphenylacetylene showed better thermal stability than conventional AlH_3_ and magnesium-doped AlH_3_, and only 0.84% of AlH_3_ decomposed at 60 °C for 48 days [71].

Flynn [72] reported a method of coating AlH_3_ with nitrocellulose. The sample material only decomposed 0.63% in 90 days at 60 °C. By coating with nitrocellulose, the compatibility of AlH_3_ with other components in some solid propellants is also significantly increased [72]. Cai et al. [73] successfully coated $\alpha -{\mathrm{AlH}}_{3}$ with fluororubber FE26 as the coating agent, and liquid CO as the anti-solvent and dispersion medium, using supercritical fluid technology. The results of differential scanning calorimetry indicate that the enthalpy of coated $\alpha -{\mathrm{AlH}}_{3}$ increases, and the thermal stability is improved. In addition, the lower electric spark sensitivity of coated $\alpha -{\mathrm{AlH}}_{3}$ shows improved applicability and stability [73].

At present, the research of coating stabilized AlH_3_ mainly focus on the screening of the coating agent, but the mechanism of coating stabilized AlH_3_ is less known. How to accurately control the coating thickness of AlH_3_ is expected to be the hinge to the next step.

## 5. Applications and Challenges

As an energetic material, AlH_3_ has extraordinary potential in the application of rocket fuel. As the power source of the rocket, solid propellant can be divided into homogeneous propellant and composite propellant [74,75]. Aluminum powder is usually used as a metal fuel to improve the energy characteristics of composite propellants [76,77]. However, the ignition temperature of aluminum powder is high, and the combustion efficiency is low [78,79,80,81]. Compared with aluminum powder, AlH_3_ has a higher energy density, which can significantly reduce the ignition temperature and produce H_2_ fuel in the combustion process, thus reducing the relative mass of combustion products [82,83,84]. In recent years, researchers have conducted extensive research on the combustion performance and compatibility of AlH_3_, and discussed the application prospect of AlH_3_ as rocket fuel [85,86,87]. Bazyn et al. [3] used shock tube and AlO emission and absorption spectroscopy to compare the combustion characteristics of AlH_3_ and Nano Al in CO_2_ and O_2_. The experimental results show that the hydrogen release time of AlH_3_ is very short. Once H_2_ is released, the combustion mode of the remaining Al is very similar to that of pure aluminum in a solid rocket motor (i.e., at a similar rate and temperature).

Il’in et al. [88] studied the combustion of AlH_3_ in the air and found that with the increase of the combustion dose of AlH_3_, the combustion products mainly changed from Al_2_O_3_ to AlN. Il’in et al. [88] also found that the combustion of AlH_3_ can be divided into three stages: (1) Far below the ignition threshold temperature of Al, the combustion stage of AlH_3_ decomposes to produce H_2_, forming a mushroom-like flame, and the flame leaves the sample surface; (2) the results show that the combustion of H_2_ is exhausted, and the flame contacts the sample, which is the low temperature combustion stage of super dispersed porous aluminum powder; (3) the high temperature combustion stage of aluminum.

Bazyn et al. [89] studied the combustion characteristics of AlH_3_ under the condition of a solid rocket motor (400–500 k). The results show that the decomposition time of AlH_3_ is exponentially correlated with temperature, which satisfies the Arrhenius type rate equation. At 1000 K, 90% of H_2_ is desorbed from AlH_3_ particles in only 38 μs. Bazyn et al. [89] also carried out a two-dimensional simulation of the aluminum particles in the propellant mixture. The results showed that AlH_3_ particles in the surface extrusion mixture decomposed to produce H_2_ before spraying from the propellant surface. The generated H_2_ will react with the binder and oxidant in the flame zone, thus affecting the stoichiometry of the zone.

DeLuca et al. [83] carried out the experiment and numerical analysis of a laboratory composite solid propellant based on AlH_3_. The ballistic performance of the AlH_3_-based propellant was evaluated experimentally and compared with the flame structure of the Al-based propellant. The experimental results show that the AlH_3_-based propellant has a first-class combustion structure, excellent ballistic and aging properties, and high quality two-phase flow. The AlH_3_-based propellant has a unique competitive advantage over the Al-based propellant.

Although AlH_3_-based rocket propellant is considered to be a promising rocket fuel, the metastability of AlH_3_ affects its practical application. AlH_3_ is easy to decompose to produce H_2_ during storage, which affects the safe storage and continuous combustion of the propellant system. At present, AlH_3_-based rocket propellant has not been applied in practice.

## 6. Conclusions

AlH_3_ is a binary metal hydride with at least seven crystal structures, among which $\alpha -{\mathrm{AlH}}_{3}$ is the most stable polymorph. During heating, $\alpha -{\mathrm{AlH}}_{3}$ will decompose into Al and H_2_, while other crystal forms of AlH_3_ (such as β—AlH_3_, γ—AlH_3_, etc.) will first transform into $\alpha -{\mathrm{AlH}}_{3}$ and then decompose into Al and H_2_. AlH_3_ is generally prepared by the reaction of LiAlH_4_ and AlCl_3_ in ether solution. Other synthetic process such as electrochemical hydrogenation, reactive milling synthesis, and regeneration with AlH_3_ adduct, have also been studied and developed.

AlH_3_ is considered as a promising hydrogen storage medium due to its high volume and weight energy density, low hydrogen release temperature, and fast reaction kinetics. AlH_3_ can be used as rocket fuel and fuel cells with high combustion heat, non-toxic, and high hydrogen content. However, the stability of AlH_3_ has become a key problem limiting its practical application (especially when used as rocket fuel). In recent years, methods to improve the stability of AlH_3_ have been widely studied, including surface passivation, doping, and surface coating. How to improve the stability of AlH_3_ without sacrificing the hydrogen release efficiency is the linchpin to the current research.

## Figures and Tables

**Table 1:** Crystallographic data of $\alpha -{\mathrm{AlH}}_{3}$ [28]. Cited from Ref. [28] with permission.

Crystal System	Trigonal
Space group	hexagonal axes
	$R\bar{3}c$(167)
Formula per unit cell, [Z]	6
Lattice parameters	A = b = 4.449 Å
	c = 11.804 Å
	α = β = 90°, γ = 120°
Density	1.477 g/cm^3^
Molecular weight	30.01 g/mol

## Data Availability

Data sharing not applicable.
